# Mentor as Sculptor, Makeover Artist, Coach, or CEO: Evaluating Contrasting Models for Mentoring Undergraduates' *Me*search Toward Publishable Research

**DOI:** 10.3389/fpsyg.2019.00231

**Published:** 2019-02-07

**Authors:** Kevin J. Holmes, Tomi-Ann Roberts

**Affiliations:** Department of Psychology, Colorado College, Colorado Springs, CO, United States

**Keywords:** mesearch, metaphors, undergraduate research mentoring, publishable research, mentoring relationship

For undergraduate students, doing original research under the guidance of an experienced scholar can be a transformative experience. Faculty-mentored undergraduate research fosters students' intellectual curiosity, reasoning and communication skills, and self-confidence, among other benefits (Lopatto, 2007; Thiry et al., 2012). Efforts to bolster institutional support for undergraduate research also trumpet its potential to advance faculty research and generate new knowledge (e.g., Elgren and Hensel, 2006). Skeptics argue, however, that the time and specialized training required to make even modest scientific contributions renders publishable research with undergraduates more aspirational than achievable (Anderson and Shore, 2008).

Here we point to an additional challenge to conducting publishable research with undergraduates, especially in psychology: the expectations that students bring to the research experience. Many students are drawn to psychology for its potential to provide insight into their own personality and experiences, fueling a desire to pursue *me*search—research about oneself (Kille, 2011). Mesearch is intrinsically rewarding to students (and to working scientists, for whom life events can be a valuable source of research inspiration; Brockman, 2004), and teaching-focused institutions such as small, residential liberal arts colleges (SLACs) often promote it. On our institution's website, for example, students are exhorted to apply for funds to “travel to India and [explore] the impacts of tourism” or to “independently study the bioethical issues of life and death” (Welcome to Colorado College, 2019). Such encouragement, coupled with students' digital-native status and the ubiquity of search engines and surveys, may give students the impression that they are already skilled at research, yet less than one-third of undergraduates show proficiency with college-level research skills such as developing a topic and distinguishing peer-reviewed articles from other sources (Library Journal, 2017). For faculty, mentoring novice researchers' mesearch endeavors while also producing publishable research of their own—both of which are expected for tenure and promotion (Volkwein and Sweitzer, 2006)—can be challenging.

Satisfying these dual demands requires a mentoring model that prioritizes both student and faculty interests in the research process. Describing such models as metaphors, as in educational psychology (e.g., educators are “gardeners” who “fertilize with interesting lectures”; McEwan, 2007), can illuminate the priorities and assumptions underlying faculty members' mentoring approaches. Two metaphors suggested in the literature—*mentor-as-sculptor* and *mentor-as-makeover-artist—*integrate students' mesearch interests and faculty research goals in resourceful ways, and thus are well-suited to accommodating these demands. Below we outline the goals and strategies guiding the two models, which we have employed successfully in our own research with undergraduates, judging from publication output and student feedback. We contrast these with two other popular models (*mentor-as-coach* and *mentor-as-CEO*), which we argue are less suited to mesearch-oriented settings because they privilege either student or faculty interests at the expense of the other.

## Our Mentoring Models: Sculptor and Makeover Artist

In the mentor-as-sculptor model (Ganser, 1999) employed by author T-A.R., students bring their mesearch ideas to a faculty mentor, who “sculpts” them into theoretically grounded, methodologically rigorous research projects. The goal in this model is publishable research that is also meaningful and rewarding to students by working with them to expand beyond Google searches to connect their mesearch ideas to existing theories (including the mentor's; e.g., Objectification Theory; Roberts et al., 2018) and empirical findings. Strategic assignments in this approach include directed readings and the creation of an annotated bibliography of sources focusing on “key words” that show students how psychological science examines a construct they might have first encountered elsewhere, such as “hegemonic masculinity” or “intersectionality.” This helps students understand how such constructs are connected to the faculty member's own theory and research program, and can now be operationalized and studied scientifically. Thus, the sculpting process can result in “molding” students' ideas into a collaboratively designed study that the mentor is willing to support, or even one the mentor had already considered but not yet developed.

In the mentor-as-makeover-artist model employed by author K.J.H., the goal is to transform, or “make over,” the mentor's research into mesearch—to show students that the questions probed in the mentor's work are relevant to their own interests. This model can work especially well when the mentor's subfield is one that undergraduates do not find immediately accessible (e.g., cognitive psychology), thus requiring some convincing that the research is mesearch-worthy. To achieve such a makeover, the mentor engages students in ongoing scientific debates (e.g., whether language shapes thought), prompting them to consider their own intuitions (e.g., perhaps based on their own language-learning experiences) in light of empirical findings (including the mentor's; e.g., Holmes et al., 2017). Through in-depth discussion with the mentor, students gain the confidence to venture a theoretical position, which they can then put to the test by taking ownership of a study in the mentor's lab. This approach, though not explicitly accorded the term “makeover” in the mentoring literature, draws on established practices for socializing students into the scholarly community (Thiry and Laursen, 2011).

Our models differ in whether they take the student's mesearch ideas or the mentor's research program as the starting point, but they also have much in common. In both, the student works collaboratively *with* the mentor and contributes valued ideas, but the mentor remains authoritative and the student still has much to learn. Both models also describe the mentoring process in artistic terms, highlighting the innovation needed to conduct quality research. To the extent that our models are discernible to students, they may communicate the view held by many scientists that “scientific research is an art form” (Wilson, 1998, p. 7) that creatively deploys the tools of a researcher's trade.

## Other Mentoring Models: Coach and CEO

Two other models carry more costs than benefits in mesearch-oriented settings, in our experience. In the mentor-as-coach model (Ganser, 1999), students are positioned as players, and the mentor's role is to support, scaffold, and cheer on their mesearch pursuits. Here the student is the one playing the research game, and winning is determined by the depth of learning and reflected in a course grade. This model may be well-suited to full professors, who have the freedom to prioritize students' learning over their own research goals. However, for earlier-career faculty, for whom publishable research is critical, the coach model leaves precious little time for their own work. Regardless of career stage, the coach model has other drawbacks. Merely “coaching” others may be viewed as a waste of doctoral training, and the research produced in this model tends to be exploratory and not immediately publishable, yet may be difficult to continue with other students, who bring different mesearch interests to be cultivated.

In another common model, mentor-as-CEO, students are positioned as workers and the goal is publishable research that advances knowledge in the mentor's subfield. Students carry out tasks in the mentor's lab, surely acquiring skills along the way, but rarely do they have the option to pursue their own mesearch interests. This model, though standard at research-intensive universities (cf. Weldon and Reyna, 2015), poses several problems for faculty at teaching-focused institutions. First, CEO mentors may have difficulty retaining student researchers, whose mesearch expectations go unfulfilled. Second, the CEO approach may not facilitate the kind of intellectual ownership provided by the best undergraduate research experiences (Lopatto, 2007), and may even be detrimental to the faculty member upon review for tenure or promotion if it is seen as falling short in promoting student learning. A final problem is that the CEO model may be challenging to embody by faculty from marginalized groups. Female faculty and faculty of color, particularly in the sciences, are consistently evaluated more negatively by students than white male faculty, reflecting implicit biases (e.g., Reid, 2010). Only those professors who fit cultural stereotypes of “brilliant, awesome, and knowledgeable,” as white male faculty do, may be seen as possessing the authority to treat students essentially as employees. Other faculty who attempt the CEO model may be seen, in contrast, as “bossy and annoying” (MacNell et al., 2015), with negative consequences for their student evaluations, research productivity, and tenure prospects.

## Challenges in Implementing Our Models

Implementing the sculptor and makeover-artist models involves some special considerations. First is the question of authorship. Unlike in the coach and CEO models (where the student and mentor, respectively, would likely be the first author), authorship is harder to determine in our models. In establishing standards, mentors might weigh several elements such as the balance of writing (who did more? did the student's writing improve over drafts?), the idea and its innovation, the time, energy, and skill devoted to collecting and analyzing data, and even the mentor's career stage (with early-career faculty facing greater pressure to produce “independent” scholarship in the form of first-authored publications). In some cases, sculptor mentors might relinquish first-authorship in the service of recognizing students' efforts to “own” the project, while makeover-artist mentors may be apt to do so only when students have demonstrated successful ownership (e.g., fluency in communicating the research). We would urge those adopting these models to communicate their criteria clearly to students and to involve students in the authorship decision-making process by inviting them to reflect on their research contributions (Fine and Kurdek, 1993).

Second, given that our models require significant intellectual ownership on the part of students, including mastery of theoretical concepts not encountered until upper-level classes, most are not ready for such responsibility until their senior year. This means that we typically work with them for only 1 year, which can go by quickly at institutions with heavy teaching loads and demands on students' time. Therefore, publishable output is often paced more slowly than in other models, and multiple students may contribute over several years (requiring careful consideration of each student's contributions when determining authorship). Another downside of the responsibility required of our students is that they may fail, make errors, or otherwise disappoint us. Faculty adopting these models must be patient with their students, fully expecting that they will not always succeed.

Third, students can develop a vexing lack of humility because of their close relationship with us and their genuine investment in the work. This can be seen in their pushing of boundaries (e.g., wishing to socialize with us off-campus) and in their behavior at conferences, where they may make bolder claims than they ought in presentations or have the temerity to demand the attention of esteemed colleagues. We have found that an earnest etiquette speech about how to comport oneself as a serious scholar both on and off campus is sometimes in order.

Finally, although we have focused on how these models operate at teaching-focused institutions like ours, faculty at research-intensive institutions who wish to prioritize student learning via our models may be unable to devote the extensive time to undergraduate training that the models demand. In such settings, graduate students who serve as undergraduate researchers' most proximal mentors might be trained to adopt the sculptor or makeover-artist model, rather than defaulting to the CEO model common at research-intensive institutions. Indeed, this “mentored mentoring” approach may be especially effective in undergraduate-targeted research programs such as the National Science Foundation's Research Experiences for Undergraduates, for which funding often hinges on the promise of substantive student involvement in publishable work (Wenzel, 2003).

Despite the risks posed by the sculptor and makeover-artist models, we believe that they yield higher rewards compared to the others. Our students enjoy the satisfaction of seeing their mesearch ideas come to fruition (sculptor model), or of discovering that ideas they once viewed as far removed from their own lives have become mesearch (makeover-artist model). When we attend conferences with our students, we have the wonderful opportunity to connect them to important researchers in our fields, and to see them display the intellectual skills and self-confidence that we helped them develop. We also find fulfillment in placing our students into excellent graduate programs that will further enhance these skills and launch their careers. Finally, of course, our models often result in published research, which not only advances our students' development and our own research goals, but also advances science.

## Author Contributions

All authors listed have made a substantial, direct and intellectual contribution to the work, and approved it for publication.

### Conflict of Interest Statement

The authors declare that the research was conducted in the absence of any commercial or financial relationships that could be construed as a potential conflict of interest.
